# Venereal Disease and the Canteen

**Published:** 1904-04

**Authors:** 


### Venereal Disease and the Canteen.

CONSIDERABLE comment has been caused by the publication of a monograph by Mr. A. C. Proffeit, concerning the British army, entitled, "Army inefficiency: It's greatest cause." The monograph deals with venereal disease as the principal reason for army inefficiency and, although the subject is an old one in so far as medical journals are concerned, Mr. Proffeit puts the entire subject in rather a new and startling light when he says that venereal diseases are responsible for one-third of the admissions to hospital. With the abolition of the contagious diseases act, the diseases in the British service increased at least 50 per cent., and *American Medicine* is authority for the statement that the increase in the American army since the abolition of the canteen is "enormous." This is quite true. And an additional fact which has apparently escaped observation is that not all of the cases of venereal disease are reported to the surgeon at an army post, for the simple reason that the enlisted man does not get credit for illness due to venereal disease. In other words it counts against him. The disability may be considered avoidable by those solicitous creatures who have taken such an active part in the abolition of the canteen with a view to the soldier's moral salvation, but it is a disability nevertheless, and because it does count against the soldier he prefers to go to a quack for treatment or to treat himself with the prescription of some druggist. Probably not more than one-fourth of the venereal disease in the army is ever reported to the surgeon, hence it is that the true figures cannot be shown in the reports of the surgeon general's office.

It is wrong to consider a soldier blameworthy because he contracts a venereal disease. He doesn't contract it wilfully any more than he deliberately contracts typhoid or any other communicable, or infectious, or contagious disease. When a man is ill he is entitled to treatment irrespective of how he contracted his illness. When the purists have closed up all the houses of ill-fame and driven their inmates broadcast throughout the cities, into furnished rooms and the back rooms of saloons, with army post canteens closed, the hymn of victory of virtue over vice is drowned in an outburst of complaint over the remarkable increase in venereal disease. The closing of the canteen was worse than a mistake; it was the kneeling of official servility before the smug-faced mask of hypocritical virtue.

